# A potent anti-inflammatory peptide from the salivary glands of horsefly

**DOI:** 10.1186/s13071-015-1149-y

**Published:** 2015-10-24

**Authors:** Lin Wei, Chunjing Huang, Hailong Yang, Min Li, Juanjuan Yang, Xue Qiao, Lixian Mu, Fei Xiong, Jing Wu, Wei Xu

**Affiliations:** Jiangsu Key Laboratory of Infection and Immunity, Institutes of Biology and Medical Sciences, Soochow University, 199 Ren-Ai Road, Suzhou Industrial Park, Suzhou, 215123 Jiangsu Province China; School of Basic Medical Sciences, Kunming Medical University, 1168 West Chunrong Road, Yuhua Avenue, Chenggong District, Kunming, 650500 Yunnan Province China; College of Biological Science and Technology, Fuzhou University, Fuzhou, 350108 Fujian China; Institute of Marine Biological Technology, School of Life Science and Biotechnology, Dalian University of Technology, Dalian, 116024 Liaoning China

**Keywords:** Horsefly, Salivary gland, Hematophagous arthropod, Cecropin, Anti-inflammation

## Abstract

**Background:**

A diverse group of physiologically active peptides/proteins are present in the salivary glands of horsefly *Tabanus yao* (Diptera, Tabanidae) that facilitate acquisition of blood meal. However, their roles in the regulation of local inflammation remains poorly understood.

**Methods:**

Induction expression profiles of immune-related molecules in the salivary glands of *T. yao* was analyzed by quantitative PCR (qPCR) after bacterial feeding. A significantly up-regulated molecule (cecropin-TY1) was selected for anti-inflammatory assay in lipopolysaccharide (LPS)-stimulated mouse peritoneal macrophages. The transcription levels of inducible NO synthase (iNOS) and pro-inflammatory cytokines were quantified by qPCR. Nitric oxide (NO) production was determined by Griess reagent. Pro-inflammatory cytokine production was determined by an enzyme-linked immunosorbent assay (ELISA). The inflammatory signals were assayed by Western blotting analysis. The secondary structure of cecropin-TY1 was measured by Circular dichroism (CD) spectroscopy. Interaction of cecropin-TY1 with LPS was evaluated by the dissociation of fluorescein isothiocyanate (FITC)-conjugated LPS aggregates and neutralization of LPS determined by a quantitative Chromogenic End-point Tachypleus amebocyte lysate (TAL) assay kit. Homology modeled structure analysis and mutation of key residues/structures were performed to understand its structure-activity relationship.

**Results:**

Cecropin-TY1 was demonstrated to possess high anti-inflammatory activity and low cytotoxicity toward mouse macrophages. In LPS-stimulated mouse peritoneal macrophage, addition of cecropin-TY1 significantly inhibited the production of nitric oxide (NO) and pro-inflammatory cytokines. Further study revealed that cecropin-TY1 inhibited inflammatory cytokine production by blocking activation of mitogen-activated protein kinases (MAPKs) and transcriptional nuclear factor-κB (NF-κB) signals. Cecropin-TY1 even interacted with LPS and neutralized LPS. The secondary structure analysis revealed that cecropin-TY1 adopted unordered structures in hydrophobic environment but converted to α-helical confirmation in membrane mimetic environments. Homology modeled structure analysis demonstrated that cecropin-TY1 adopted two α-helices (Leu3-Thr24, Ile27-Leu38) linked by a hinge (Leu25-Pro26) and the structure surface was partly positively charged. Structure-activity relationship analysis indicated that several key residues/structures are crucial for its anti-inflammatory activity including α-helices, aromatic residue Trp2, positively charged residues Lys and Arg, hinge residue Pro26 and N-terminal amidation.

**Conclusions:**

We found a novel anti-inflammatory function of horsefly-derived cecropin-TY1 peptide, laying groundwork for better understanding the ectoparasite-host interaction of horsefly with host and highlighting its potency in anti-inflammatory therapy for sepsis and endotoxin shock caused by Gram-negative bacterial infections.

**Electronic supplementary material:**

The online version of this article (doi:10.1186/s13071-015-1149-y) contains supplementary material, which is available to authorized users.

## Background

Hematophagous arthropods have evolved effective mechanisms to suppress their host’s hemostatic system and immune response to get a blood meal successfully. Their salivary glands can produce a wide array of active compounds including antihemostatic and immunoregulatory substances [1–9]. A majority of antihemostatic compounds have been identified and can be divided into several categories including inhibitors of coagulation factors (Factors VII, V, IIa, and Xa) and platelet functions, fibrin(ogen)olytic enzymes, and vasoactive peptides [3–5]. Immunoregulatory factors from arthropods especially from ticks have been extensively investigated [10–15]. These immunoregulatory factors such as immunosuppressive peptides and antimicrobial peptides (AMPs) are crucial for blood-sucking arthropods to suppress the immune response including innate immunity, adaptive immunity and inflammation [5, 14–17]. Horseflies are economically important blood-feeding insects and vectors for pathogens such as filariasis [5]. There have been reported a diverse group of active compounds in the salivary glands of horseflies like other hematophagous arthropods including mosquitoes [1], flies [2], and ticks [10, 12]. Although the antihemostaic substances in horsefly have been extensively exploited in previous work [5, 18, 19], comparatively few investigations on the anti-inflammatory effects of horsefly-derived AMPs were conducted.

AMPs are small gene-encoded defensive effectors and play key roles in the innate immunity in all living organisms. Insects are an important source of AMPs [20]. Since the first observation of antimicrobial activity in the hemolymph of bacteria-challenged pupae of the giant silk moths (*Samia Cynthia* and *Hyalophora cecropia*) in 1974 and the first purification of AMP from the hemolymph of *H. cecropia* in 1980, over 200 AMPs have been identified or purified from insects [20]. Generally, insect AMPs are comprised of four groups based on their structural motifs and unique sequences. They are (i) α-helical peptides (cecropin and moricin), (ii) cysteine-rich peptides with intramolecular cysteine disulfide bonds forming hairpin-like β-sheets or α-helical/β-sheet mixed structures (insect defensin and drosomycin), (iii) proline-rich peptides (apidaecin, drosocin, and lebocin), and (iv) glycine-rich peptides/proteins (attacin and gloverin) [20].

Among these insect-derived AMPs, cecropins constitute a large family of cationic α-helical peptides composed of 35–39 amino acids, and most of them are amidated at the C-terminus [20]. Cecropin was the first insect AMP purified from the hemolymph of *H. cecropia* in 1980 [21]. Since then, a variety of insect-derived cecropin AMPs were identified in lepidopteran, dipteran, and coleopteran [20]. Cecropins usually have broad antimicrobial spectrum against various microorganisms including Gram-positive and Gram-negative bacteria [22, 23], fungi [24, 25], parasites [26, 27] and HIV-1 virus [28]. In addition to antimicrobial activity, several cecropins showed strong anti-inflammatory activity in LPS-stimulated macrophages through interaction with LPS on the basis of their α-helical structures [29, 30]. Cecropins usually adopted random coil conformations in aqueous solutions. While these unordered structures converted to amphipathic α-helical structures in the membrane-like environments to exert its biological activity [20, 29, 30].

Recently, a wide array of physiologically active molecules such as antihemostatic substances (fibrin(ogen)olytic, Arg-Gly-Asp-motif containing proteins, vasodilator peptides, etc.), immunosuppressive peptides (tabimmuregulins), AMPs (attactin, defensin and cecropin) and allergens (Tab a 1, Tab a 2, Tab y 1) have been identified in the salivary glands in horsefly of *T. yao* [3, 5, 31–35]. To identify whether there are anti-inflammatory agents in the salivary glands of horsefly *T. yao*, induction expression of immune-related genes (immunosuppressive peptides and AMPs) in their salivary glands were analyzed after bacterial feeding. It was demonstrated that cecropin-TY1 expression was dramatically up-regulated among immune-related genes. Cecropin-TY1 was previously identified as an AMP with antimicrobial activity in the salivary glands of *T. yao* [5]. In the current work, cecropin-TY1 showed strong anti-inflammatory effects in LPS-stimulated mouse peritoneal macrophages and low cytotoxicity. The effects of cecropin-TY1 on LPS-activated inflammatory signaling and the interaction of cecropin-TY1 with LPS were investigated. The secondary structures of cecropin-TY1 in different solutions were exploited by CD spectra and the 3D structures were homology modeled to understand the interaction between cecropin-TY1 and LPS. Anti-inflammatory effects of the derivatives of cecropin-TY1 were also investigated to understand the key residues/structures for its anti-inflammatory activity.

## Methods

### Induction expression analysis

Horseflies were collected from Shanxi province as previously described [3, 5]. The collected *T. yao* (~800 flies) were randomly grouped in two cages (100 × 80 × 60 cm) covered with grenadine and kept at temperature of 25 ± 2 °C, humidity of 80–90 % and a 12 h/12 h photoperiod. After a 12-h starvation, horseflies were fed with fresh chicken blood containing 1 % sodium citrate (w/v) supplemented with Gram-negative bacteria *Escherichia coli* ATCC 8739 (2 × 10^6^/mL) [36]. The control group was fed with the same chicken blood without bacteria. The salivary glands of horseflies were dissected under a microscope at 0, 6, 12, 24, 36, 48 and 72 h after blood meal. The salivary gland of each horsefly was excised in phosphate buffer solution (100 mM, pH 6.0) on ice immediately [3, 5]. Total RNA extraction was performed by Trizol reagent (Life Tech, USA) according to the kit instruction. cDNA was synthesized with PrimeScript® Reverse Transcriptase Kit (Takara, Japan). The induction expression of immune-related genes including tabimmuregulins and AMPs was analyzed by qPCR to screen anti-inflammatory agents in the salivary glands of horseflies as described in qPCR section.

### Ethical approval

The study was approved by the Animal Care and Use Ethics Committee of Kunming Medical University.

### Quantitative PCR

qPCR was performed using SYBR green master mix (Takara, Japan) on a Realplex Mastercycler real-time PCR system (Eppendorf, Germany) according to the manufacturer’s instruction. The gene transcription levels were normalized to GAPDH or β-actinas as illustrated in figure legends and calculated by ΔΔCt method. The accuracy of qPCR results were checked by melting curve analysis. qPCR primers were listed in Additional file 1: Table S1.

### Peptide synthesis

Cecropin-TY1 and its derivatives (disruption of α-helices, mutation of aromatic residue Trp2, mutation of positively charged residues, deletion of hinge residue Pro26 and mutation of N-terminal amidation) were synthesized for anti-inflammatory effects investigation as listed in Table 1. The peptides were synthesized by solid phase synthesis on an Applied Biosystems model 433A peptide synthesizer by GL Biochem (Shanghai) Ltd. (Shanghai, China). The synthetic peptides were purified by high-performance liquid chromatography. The purified peptides were subjected to an automated Edman degradation protein sequencer to confirm the accuracy of amino acid sequence, and MALDI-TOF mass spectrometry to confirm the purity > 98 %.

**Table 1:** The amino acid sequence of the derivatives of cecropin-TY1

Mutant name	Amino acid sequence	Mutation site^a^
N39 mutant	GWLKKIGKKIERVGQNVRNAAISTLPIAQGAAGVAGALN-COOH	N^39^CONH_2_ → N^39^COOH
W2 mutant	GALKKIGKKIERVGQNVRNAAISTLPIAQGAAGVAGALN-NH_2_	W^2^ → A^2^
P26 mutant	GWLKKIGKKIERVGQNVRNAAISTL_IAQGAAGVAGALN-NH_2_	lacked the hinge residue P^26^
KR mutant	GWLAAIGAAIEAVGQNVANAAISTLPIAQGAAGVAGALN-NH_2_	K^4^ → A^4^, K^5^ → A^5^, K^8^ → A^8^, K^9^ → A^9^, R^12^ → A^12^, R^18^ → A^18^
scr-cec-TY1	RGQANILAGKNIKIRSGAAAGVGKTPQKANVEVLALGIW-NH_2_	Scrambled cecropin-TY1, disrupted the helices

^a^The mutant sites were underlined

### Cytotoxicity assay

Mouse peritoneal macrophages were prepared according to previous method [37]. Brewer thioglycollate medium (3 %, w/v, Sigma-Aldrich, USA) was intraperitoneally injected to C57BL/6 mouse. After 3 days, the mouse was euthanized and intraperitoneally injected with 20 mL RPMI-1640 medium to collect peritoneal macrophages. RAW264.7 cells were cultured in RPMI 1640 medium (10 % FBS, 100 U/mL penicillin and 100 μg/mL streptomycin, Gibco, USA) at 37 °C humidified with 5 % CO_2_. Mouse macrophages, peritoneal macrophages and RAW264.7 cells, were seeded in a 96-well (2 × 10^4^ cells/well) plate, and cultured in RPMI 1640 medium (100 μL) supplemented with 2 % FBS, 100 U/mL penicillin and 100 μg/mL streptomycin (Gibco, USA) at 37 °C in a humidified 5 % CO_2_ incubator. The cytotoxicity of cecropin-TY1 against mouse macrophages was determined by Cell Counting Kit-8 assay (CCK-8) following the kit instruction. In brief, serial 2-fold peptide dilutions were added to each well, and control wells received the same volume of phosphate-buffered solution (PBS, 8 g/L NaCl, 0.2 g/L KCl, 0.2 g/L KH_2_PO_4_, 2.89 g/L Na_2_HPO_4_ · 12H_2_O, pH 7.4). After 24-h incubation, CCK-8 solution (10 μL/well) was added and incubated for an additional 4 h. The absorbance at 450 nm was monitored on a microplate reader (Epoch Etock, BioTek, USA).

### Detection of NO production in macrophages

Peritoneal macrophages were seeded in two 24-well plates (2.5 × 10^5^ cells/well) and cultured in RPMI-1640 containing 2 % FBS, 100 U/mL penicillin, and 100 μg/mL streptomycin (Gibco, USA). Cells were incubated with peptides (0, 5, 10, and 20 μg/mL) in the presence or absence of LPS (100 ng/mL, from *Escherichia coli* 0111:B4, Sigma-Aldrich, USA). After 6-h incubation, cells in one plate were washed twice with ice-cold PBS and lysed by Trizol reagent (Life Tech, USA) for total RNA extraction. PrimeScript® Reverse Transcriptase Kit (Takara, Japan) was used to synthesize cDNA for qPCR to examine the transcription levels of inducible nitric oxide synthase (iNOS), which is necessary for NO production [38]. After 24-h incubation, culture medium of each wells of another plate was harvested for nitrite detection, which indirectly reflected the NO production [39, 40]. Nitrite accumulation levels were determined by NO detection kit (Beyotime, China) following the kit instruction.

### Cytokine production analysis by qPCR and ELISA

Peritoneal macrophages were cultured in a 24-well (2.5 × 10^5^/well) plate in RPMI-1640 containing 2 % FBS, 100 U/mL penicillin, and 100 μg/mL streptomycin (Gibco, USA). Cells were incubated with peptides (0, 5, 10, and 20 μg/mL) with or without 100 ng/mL LPS (from *E. coli* 0111:B4, Sigma-Aldrich, USA) for 6 h. After treatment, culture medium was collected for TNF-α, IL-1β and IL-6 quantification using enzyme-linked immunosorbent assay (ELISA) kits (Dakewei, China). Cells were washed with ice-cold PBS and lysed by Trizol reagent (Life Tech, USA). Total RNA was extracted for cDNA synthesis to quantify TNF-α, IL-1β and IL-6 transcription levels by qPCR as described in qPCR section.

### Western blot analysis

Peritoneal macrophages were seeded in a 6-well (2 × 10^6^/well) plate and cultured in RPMI-1640 supplemented with 2 % FBS, 100 U/mL penicillin, and 100 μg/mL streptomycin (Gibco, USA). Cells were treated with different concentrations of cecropin-TY1 (0, 5, 10, and 20 μg/mL) in the presence or absence of 100 ng/mL LPS (from *E. coli* 0111:B4, Sigma-Aldrich, USA). After 30-min incubation, cells were washed twice with ice-cold PBS and lysed with RIPA lysis buffer (Beyotime, China) on ice for 30 min according to our previous method [14]. Protein concentration was quantified by BCA Protein Assay Kit (Thermo, Germany). About 40 μg protein was separated on a 10 % SDS-PAGE gel and transferred to a polyvinylidene difluoride membrane. The membrane was blocked by incubating with 5 % BSA (BD, USA) dissolved in Tris-buffered solution Tween-20 (TBST, 2.42 g/L Trisbase, 8 g/L NaCl, 0.1 % Tween 20, pH 7.6) for 2 h at room temperature. The membrane was then incubated with primary antibodies against ERK, phospho-ERK, p38, phospho-p38, JNK, phospho-JNK, phospho-IκBα, NF-κB p65 and phospho-NF-κB p65 (1:2000, Cell Signaling Technology, USA) and GAPDH/β-actin (1:5000, Santa Cruz Biotechnology, USA) overnight at 4 °C, respectively. After incubation, the immunoblot was washed three times with TBST for 5 min each time, and incubated with secondary antibody (1:5000, Cell Signaling Technology, USA) at room temperature for 1 h. Signals were measured by enhanced chemiluminescence kit in a dark room after washing three times with TBST for 10 min each time (TIANGEN, China).

### Interaction between cecropin-TY1 and LPS

Fluorescein isothiocyanate (FITC)-conjugated LPS (1 μg/mL, Sigma-Aldrich, USA) was excited at 480 nm and monitored the changes in the emission of FITC-LPS at 515 nm in the incubation of different concentrations of peptides (0, 12.5, 25, 50, 100 μg/mL). Peptides were dissolved in 10 mM phosphate buffer at pH 6.0. The interactions between peptides and LPS were further assessed by a quantitative Chromogenic End-point Tachypleus amebocyte lysate (TAL) assay kit (Xiamen Houshiji, China) following the kit instruction. Briefly, different concentrations of peptides (0, 12.5, 25, 50, 100 μg/mL) were incubated with LPS (1 μg/mL, Sigma-Aldrich, USA) at 37 °C for 30 min. After incubation, 100 μL TAL solution was added to 100 μL LPS-peptide mixtures in a pyrogen-free tube and incubated at 37 °C for 10 min. Then, LPS-peptide mixtures were added with pre-warmed substrate solution for additional 6-min incubation at 37 °C. Lastly, the absorbance at 545 nm was measured on a microplate reader (Epoch Etock, BioTek, USA). The percentages of LPS-neutralizing activity were calculated.

### Circular dichroism analysis

CD spectra were collected on a Jasco-810 spectropolarimeter (Jasco, Tokyo, Japan) with a 1-mm path-length cell at 25 °C and 0.2-nm intervals from 190 to 260 nm. Cecropin-TY1 was dissolved in H_2_O, TFE/water solution, SDS/water solution and LPS/water solution at the concentration of 0.2 mg/mL. The data from three scans were averaged and smoothed using the Jasco-810 software for each spectrum. CD data were expressed as the mean residue ellipticity (θ) in deg.cm^2^.dmol^−1^.

### Structure modeling analysis

Three-dimensional (3D) structure of cecropin-TY1 was modeled by Easymodeller version 2.0. The solution NMR structures of papiliocin (59 % identity, PDB entry code 2LA2) from swallowtail butterfly of *Papilio xuthus* was selected as the template for homology modeling. The comparative 3D structure model of cecropin-TY1 was optimized using MODELLER and visualized using PYMOL software (http://www.pymol.org/) [7].

### Statistical analysis

Statistical analysis was performed using GraphPad Prism 5.0 (GraphPad Software Inc., San Diego, CA, USA) and Stata 10.0 software (Stata Corporation, College Station, TX, USA). Data were presented as mean ± SEM, and compared using two-tailed equal variance Student’s *t*-test. *P < 0.05 and **P < 0.01 were considered as statistical significance.

## Results

### Induction expression of cecropin-TY1 in the salivary glands of *T. yao*

**Fig. 1:**
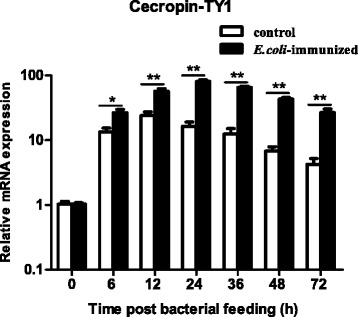
Induction expression of cecropin-TY1 in the salivary glands of horsefly *T. yao*. The transcription levels of cecropin-TY1 at different time course were normalized to β-actin. Induction expression levels of cecropin-TY1 in the salivary glands after bacterial feeding were calculated relative to the level of that in control group at 0 h, which was arbitrarily defined as 1. At 6, 12, 24, 36, 48 and 72 h post feeding, the fold increase of control groups are 13.4, 24.1, 16.4, 12.4, 6.8 and 4.24, and the fold increase of bacterially challenged groups are 26.6, 57.1, 80.5, 65.3, 43.3 and 26.8, respectively. Data were presented as mean ± SEM. *P <0.05, **P < 0.01, values of bacterial feeding groups are significantly different from control groups at different time points as indicated (*n* = 40)

Release of LPS during Gram-negative bacteria infection can induce strong inflammatory response [41]. To screen anti-inflammatory molecules, the induction expression of immune-related molecules were analyzed in the salivary glands of *T. yao* after bacterial feeding. By analysis of the transcription of tabimmuregulins and AMPs in the salivary glands, most of them were up-regulated (data not shown). Among these up-regulated innate immunity molecules, cecropin-TY1 was dramatically up-regulated as shown in Fig. 1. At 6, 12, 24, 36, 48 and 72 h post bacterial feeding, the transcription levels of cecproin-TY1 increased by 2.0, 2.4, 4.9, 5.3, 6.4, and 6.3 fold compared to the control group, respectively (Fig. 1). Cecropin-TY1 (GenBank accession No. ABX80069.1) was previously identified as a member of cecropin AMPs with known mature peptide amino acid sequence, molecular weight, net charges and theoretical pI as listed in Additional file 1: Table S2 [5].

### Cecropin-TY1 is non-toxic to mouse macrophages

In order to evaluate the effects of cecropin-TY1 on mouse macrophages, the cell viability was determined in the presence of serial concentrations of peptides incubated with peritoneal macrophages and RAW264.7 cells. At the concentration as high as 200 μg/mL, cecropin-TY1 didn’t show any cytotoxicity against mouse peritoneal macrophages and RAW264.7 cells.

### Inhibition of LPS-induced NO production

**Fig. 2:**
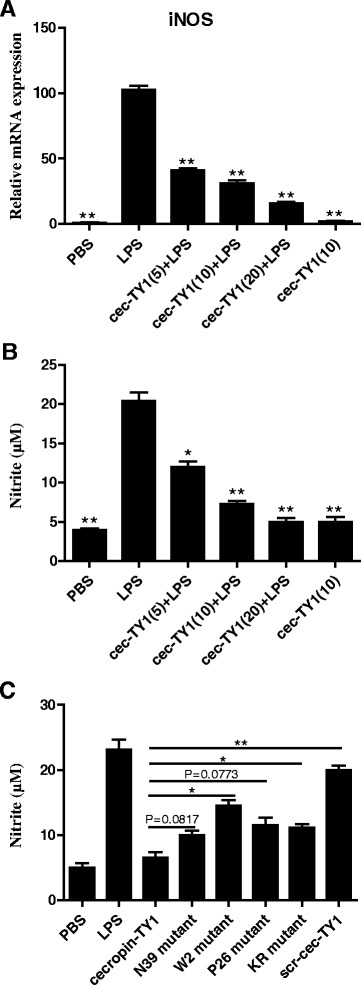
Inhibitory effects of cecropin-TY1 on LPS-stimulated NO production in mouse peritoneal macrophages. **a** Inhibitory effects of cecropin-TY1 on LPS-stimulated iNOS mRNA transcription in peritoneal macrophages. The transcription levels of iNOS in peritoneal macrophages after different treatment were normalized to GAPDH. The transcription level of iNOS in macrophages induced by 100 ng/mL LPS was arbitrarily defined as 100 %. Data were presented as mean ± SEM. *P <0.05, **P < 0.01, values of peptide-treated groups are significantly different from that induced by 100 ng/mL LPS alone. **b** Inhibitory effects of cecropin-TY1 on LPS-stimulated nitrite production in peritoneal macrophages. Peritoneal macrophages were stimulated with or without LPS (100 ng/mL), then different concentrations of cecropin-TY1 (cec-TY1, 5, 10, 20 μg/mL) were added as indicated and incubated for 6 h to detect iNOS transcription levels and 24 h to detect nitrite accumulation levels, respectively. Data were presented as mean ± SEM. *P <0.05, **P < 0.01, values of peptide-treated groups are significantly different from that induced by 100 ng/mL LPS alone. **c** Effects of different derivatives of cecropin-TY1 on LPS-stimulated nitrite production. Peritoneal macrophages were stimulated with 100 ng/mL LPS, then peptides (5 μM) were added as indicated and incubated for 24 h to detect nitrite accumulation levels in the culture medium. Data were presented as mean ± SEM. *P <0.05, **P < 0.01, values of derivative-treated groups are significantly different from that of cecropin-TY1-treated group

To determine the effect of cecropin-TY1 on LPS-induced NO production, iNOS transcription and nitrite production upon LPS stimulation in the presence or absence of cecropin-TY1 were detected. 100 ng/mL LPS significantly induced iNOS transcription, and cecropin-TY1 inhibited LPS-activated iNOS transcription in a dose-dependent manner (Fig. 2a). At the concentration of 5, 10, and 20 μg/mL, cecropin-TY1 attenuated 58.8, 68.7 and 84.2 % iNOS transcription, respectively. iNOS is the synthase required for NO production [38]. So we next detected the nitrite accumulation in culture medium. As shown in Fig. 2b, LPS (100 ng/mL) induced about 20.4 μM nitrite production, and cecropin-TY1 attenuated nitrite production in a dose-dependent manner. At the concentration of 5, 10, and 20 μg/mL, cecropin-TY1 attenuated about 40.0, 63.7 and 75.0 % LPS-induced nitrite production, respectively. The derivatives of cecropin-TY1 showed reduced inhibitory effects on LPS-induced nitrite production as shown in Fig. 2c.

### Inhibition of LPS-induced pro-inflammatory cytokine production

**Fig. 3:**
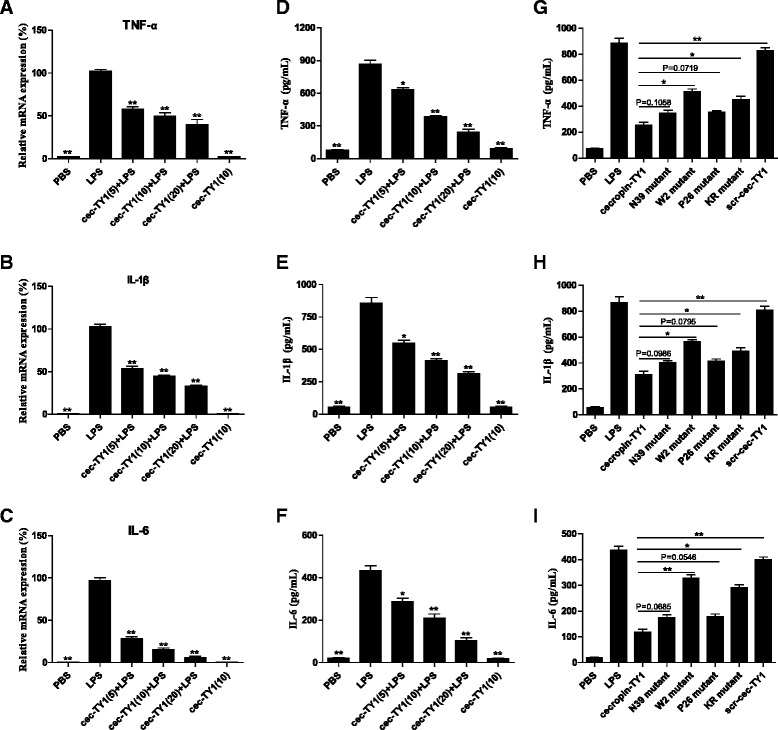
Inhibitory effects of cecropin-TY1 on LPS-stimulated pro-inflammatory cytokines production in mouse peritoneal macrophages. **a**–**c** Inhibitory effects of cecropin-TY1 on LPS-stimulated (**a**) TNF-α, (**b**) IL-1β and (**c**) IL-6 transcription. The transcription levels of pro-inflammatory cytokines in peritoneal macrophages after different treatment were normalized to GAPDH. The transcription levels of pro-inflammatory cytokine in macrophages induced by 100 ng/mL LPS were arbitrarily defined as 100 %. Data were presented as mean ± SEM. *P <0.05, **P < 0.01, values of peptide-treated groups are significantly different from that induced by 100 ng/mL LPS alone. **d**–**f** Inhibitory effects of cecropin-TY1 on LPS-stimulated (**d**) TNF-α, (**e**) IL-1β and **(f)** IL-6 production. Peritoneal macrophages were stimulated with or without LPS (100 ng/mL), then different concentrations of cecropin-TY1 (cec-TY1, 5, 10, 20 μg/mL) were added as indicated and incubated for 6 h to detect pro-inflammatory cytokine transcription and production. Data were presented as mean ± SEM. *P <0.05, **P < 0.01, values of peptide-treated groups are significantly different from that induced by 100 ng/mL LPS alone. **g**–**i** Effects of the derivatives of cecropin-TY1 on LPS-stimulated (**g**) TNF-α, (**h**) IL-1β and (**i**) IL-6 production. Peritoneal macrophages were stimulated with 100 ng/mL LPS, then peptides (5 μM) were added and incubated for 6 h to detect pro-inflammatory cytokine production. Data were presented as mean ± SEM. *P <0.05, **P < 0.01, values of derivative-treated groups are significantly different from that of cecropin-TY1-treated group

To determine the effects of cecropin-TY1 on the production of pro-inflammatory cytokines, we tested the transcription and production of LPS-induced pro-inflammatory cytokines including TNF-α, IL-1β and IL-6 in peritoneal macrophages in the presence or absence of cecropin-TY1 as illustrated in Fig. 3. LPS (100 ng/mL) alone significantly activated the transcription and production of TNF-α, IL-1β and IL-6, and cecropin-TY1 significantly reduced the transcription and production of these three pro-inflammatory cytokines in a dose-dependent manner. At the concentration of 20 μg/mL, cecropin-TY1 attenuated LPS-stimulated TNF-α, IL-1β and IL-6 transcription by 60.3, 67.5 and 94.1 %, respectively (Fig. 3a–c). As a result, 20 μg/mL of cecropin-TY1 subsequently reduced TNF-α, IL-1β and IL-6 production by 72.1, 63.4 and 76.2 % induced by LPS, respectively (Fig. 3d–e). The derivatives were less active than cecropin-TY1 on LPS-induced inflammatory cytokine production (Fig. 3g–i).

### Inhibition of LPS-induced inflammatory signal pathways

**Fig. 4:**
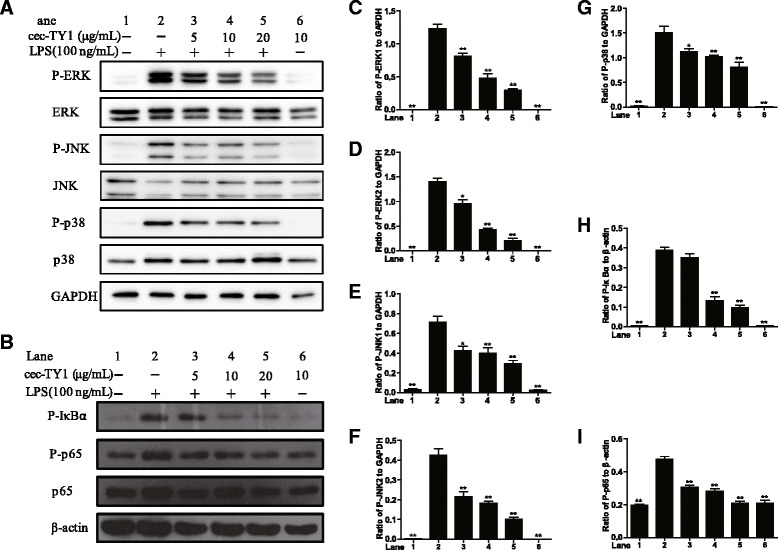
Inhibitory effects of cecropin-TY1 on LPS-stimulated inflammatory response signal pathway in peritoneal macrophages. **a**–**b** Effects of cecropin-TY1 on (**a**) MAPKs and (**b**) NF-κB activation in peritoneal macrophages after 100 ng/mL LPS stimulation. The photograph is a representative one of three independent experiments. Peritoneal macrophages were stimulated with or without LPS (100 ng/mL), then different concentrations of cecropin-TY1 (cec-TY1, 5, 10, 20 μg/mL) were added immediately and incubated for 30 min, and macrophages were lysed for western blot analysis. (**c**–**i** Ratio of (**c**) P-ERK1 (42 kDa), (**d**) P-ERK2 (44 kDa), (**e**) P-JNK1 (46 kDa), (**f**) P-JNK2 (54 kDa), (**g**) P-p38, (**h**) P-IκBα and (**i**) P-p65 to β-actin. Band densities were analyzed using Quantity One software (Bio-Rad, Richmond, CA USA). Data were presented as mean ± SEM. *P <0.05, **P < 0.01, ratios of peptide-treated groups are significantly different from that induced by 100 ng/mL LPS alone

To address the anti-inflammatory mechanisms of cecropin-TY1 on LPS-stimulated mouse peritoneal macrophages, the effects of cecropin-TY1 on LPS-induced inflammatory signal pathways were investigated. As illustrated in Fig. 4, LPS (100 ng/mL) significantly activated MAPKs and NF-κB signal pathways. Cecropin-TY1 significantly blocked the activation of MAPKs and NF-κB signal pathways in a dose-dependent manner. At the concentration of 20 μg/mL, cecropin-TY1 inhibited 75.5 % P-ERK1, 84.9 % P-ERK2, 61.9 % P-JNK1, 66.1 % P-JNK2, 46.4 % P-p38, 74.8 % P-IκBα and 55.9 % P-p65 expression induced by LPS, respectively.

### Cecropin-TY1 interacts with LPS and neutralizes its activity

**Fig. 5:**
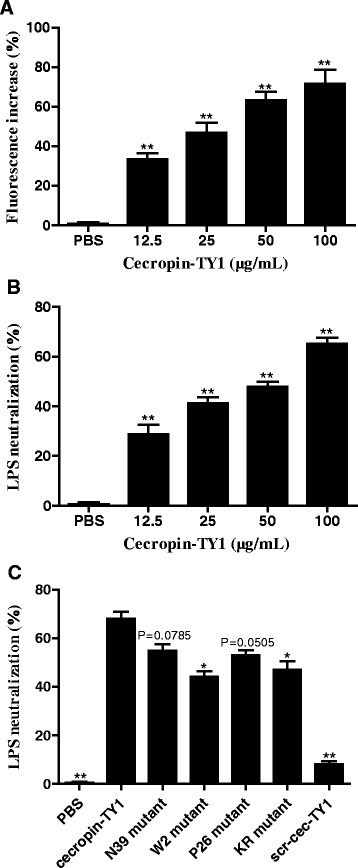
The interaction between cecropin-TY1 and LPS. **a** Enhancement of the intensity of FITC-labeled LPS at different concentrations of cecropin-TY1 as indicated. Data were presented as mean ± SEM. *P <0.05, **P < 0.01, values of peptide-treated groups are significantly different from that of PBS-treated group. **b** Cecropin-TY1 neutralized LPS at different concentrations as indicated. LPS-neutralizing activity of cecropin-TY1 was determined by TAL assay kit. Data were presented as mean ± SEM. *P <0.05, **P < 0.01, values of peptide-treated groups are significantly different from that of PBS-treated group. **c** LPS-neutralization activity of cecropin-TY1 and its derivatives (5 μM). Data were presented as mean ± SEM. *P <0.05, **P < 0.01, values of derivative-treated groups are significantly different from that of cecropin-TY1-treated group

To address the underlying anti-inflammatory mechanism of cecropin-TY1 in LPS-stimulated peritoneal macrophages, the interaction between cecropin-TY1 and LPS was investigated. Interaction of AMPs with LPS can be measured as an increase in fluorescence of FITC-conjugated LPS, which indirectly reflects the dissociation of large LPS aggregates into smaller sizes [30, 42, 43]. As shown in Fig. 5a, the addition of cecropin-TY1 caused a dose-dependent increase of FITC-LPS fluorescence, indicating that the interaction of cecropin-TY1 with LPS resulted in the dissociation of LPS aggregates. At the concentration of 12.5, 25, 50 and 100 μg/mL, cecropin-TY1 increased 33.4, 46.7, 63.3, and 71.6 % fluorescence, respectively. The interaction of cecropin-TY1 with LPS was further evaluated by TAL assay for LPS-neutralization activity. Cecropin-TY1 neutralized LPS in a dose-dependent manner (Fig. 5b). At the concentration of 12.5, 25, 50 and 100 μg/mL, cecropin-TY1 neutralized 28.8, 41.3, 47.9 and 65.1 % LPS, respectively. The LPS-neutralizing activity of cecropin-TY1 is more potent than that of derivatives (Fig. 5c).

### Secondary structures of cecropin-TY1

**Fig. 6:**
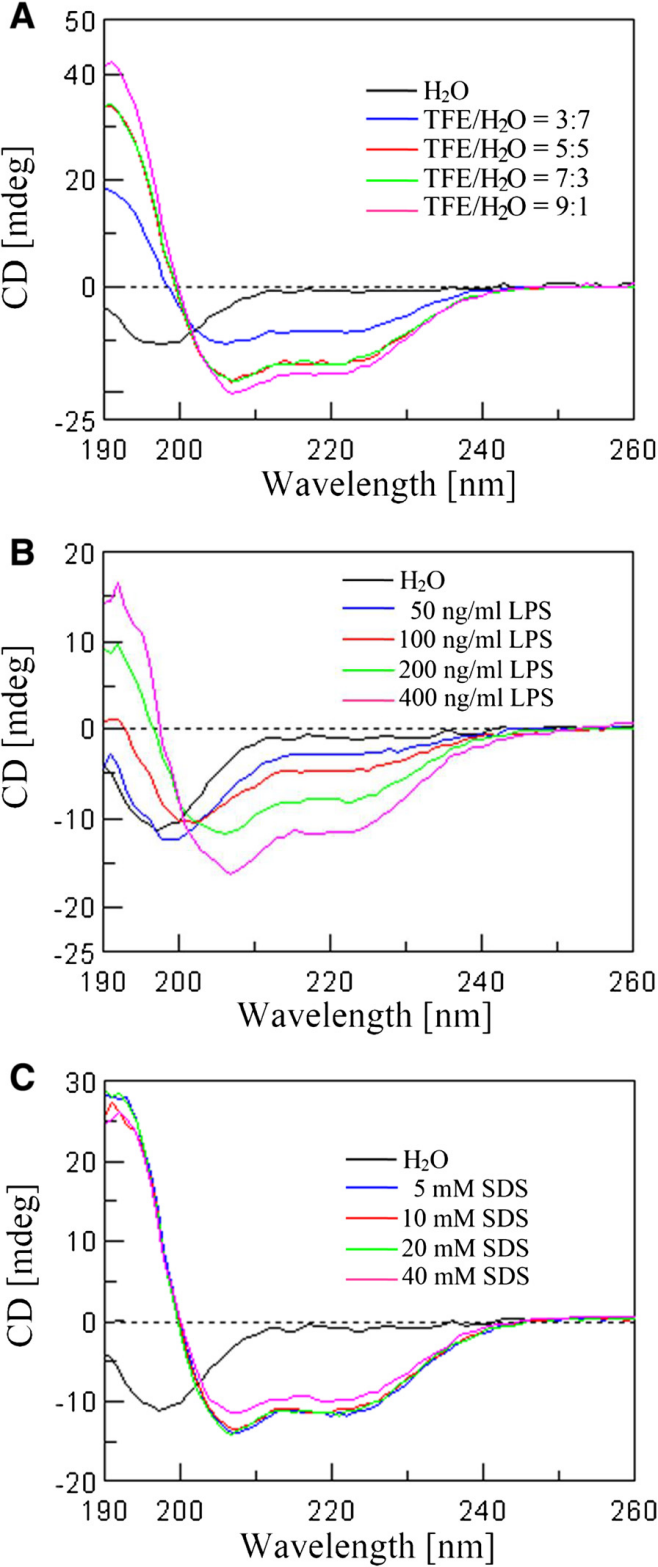
CD spectra of cecropin-TY1 in different solutions. **a** TFE/H_2_O solution. **b** LPS/H_2_O solution. **c** SDS/H_2_O solution. TFE, LPS and SDS solutions were prepared as indicated concentrations. Cecropin-TY1 was dissolved in H_2_O, TFE/water solution, LPS/water solution and SDS/water solution at the concentration of 0.2 mg/mL

To assess the secondary structures of cecropin-TY1 in membrane mimetic environments, the CD spectra of cecropin-TY1 in different membrane-like solutions were recorded and analyzed as shown in Fig. 6 and Table 2. The CD spectra of cecropin-TY1 dissolved in H_2_O showed a strong negative absorption at 199 nm, demonstrating that cecropin-TY1 mainly adopted random coil structures in aqueous solution. The conformations exhibited changes in TFE/H_2_O solutions, LPS micelles and SDS micelles. In these membrane-like environments, the CD spectra exhibited double negative absorptions at 208 and 222 nm, indicating that cecropin-TY1 adopted a significant degree of α-helical conformations in membrane-like solutions (Fig. 6). In aqueous solution, the secondary structures of cecropin-TY1 are composed of 71.1 % random coil conformation without α-helical conformation. While in TFE/H_2_O (9:1), LPS/H_2_O (400 ng/mL) and SDS/H_2_O (40 mM) solutions, the α-helical structures increased to 45.1, 14.8 and 54.4 %, respectively (Table 2).

**Table 2:** Secondary structural components of cecropin-TY1 in different solutions

Solution	Helix (%)^a^	Beta (%)^a^	Turn (%)^a^	Random (%)^a^
H_2_O	0.0	0.0	28.9	71.1
TFE/H_2_O				
3:7	28.4	28.7	0.0	42.8
5:5	36.9	22.4	0.0	40.7
7:3	37.8	22.4	0.0	39.9
9:1	45.1	15.0	0.0	39.9
LPS (ng/mL)				
50	0.0	32.4	12.4	55.2
100	2.9	46.6	3.1	47.4
200	12.7	44.5	1.7	41.1
400	14.8	47.8	0.1	37.2
SDS (mM)				
5	45.3	17.5	0.0	37.2
10	41.2	22.7	0.0	36.1
20	46.6	13.4	0.0	40.0
40	54.4	7.4	0.0	38.2

^a^Jasco-810 software was used to deconvolute CD spectra into fractional contents and these data are the average value of three scans

### Homology modeled structure analysis of cecropin-TY1

**Fig. 7:**
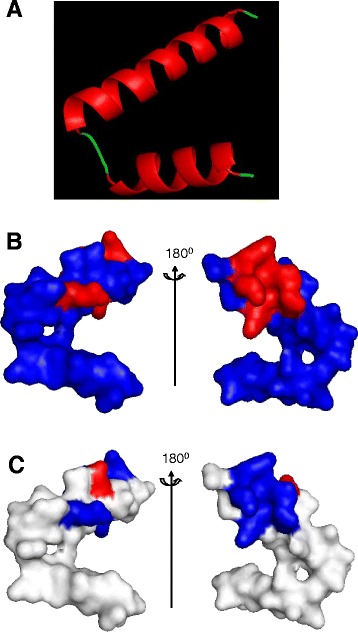
Homology structure analysis of cecropin-TY1. **a** Representative solution structure of cecropin-TY1 derived from homology modeling. It mainly consists of two α-helical regions (in red) in residues Leu3-Thr24 and Ile27-Leu38 linked by a hinge (in green) in residues Leu25-Pro26. **b** Basic amino acid residues (Lys and Arg, in red) are displayed in the structures. **c** Electrostatic surface of cecropin-TY1 which takes the same orientation as that in the (A), positively charged region and negatively charged region are shown in blue and red, respectively. Homology modeled structure analysis was performed by Easymodeller version 2.0 and optimized by using PYMOL software

The amphiphilic structure of helix and electrostatic surface of such host defensive peptides are important for their interactions with LPS [30, 40]. To investigate the structural basis of cecropin-TY1, the homology modeled structures were analyzed as shown in Fig. 7. It exhibited a helix-hinge-helix confirmation, suggesting the presence of an α-helix (in red) from residues Leu3 to Thr24 linked by a hinge region to another α-helix (in red) from Ile27 to Leu38 (Fig. 7a). The amphiphilic α-helix is the predominant structural feature for cecropin-TY1. It also showed that the 6 basic residues (4 Lys and 2 Arg), which have positive charges, were partly distributed in the surface of 3D structure (Fig. 7b, in red). Electrostatic surface analysis revealed that most regions of the electrostatic surface are positively charged (Fig. 7c in blue).

## Discussion

Horseflies have been used as anti-thrombosis materials for hundreds of years in China and some other eastern countries [3]. Recently, a variety of physiologically active compounds have been identified in the salivary glands of horsefly, *T. yao*. They are (i) fibrin(ogen)olytic enzymes, (ii) Arg-Gly-Asp-motif containing proteins, (iii) a serine protease inhibitor, (iv) a serine protease, (v) a protease, (vi) a apyrase, (vii) vasodilator peptides, (viii) a peroxidase, (ix) two metallothioneins, (x) a hyaluronidase, (xi) immunosuppressive peptides and (xii) three family of AMPs [3, 5, 31–35]. All these active compounds can be generally classified into several groups such as antihemostatic factors, immunosuppressive factors, antimicrobial factors and allergens. But anti-inflammatory agents were poorly understood in the salivary glands of *T. yao*.

In the present work, a novel anti-inflammatory molecule (cecropin-TY1) was identified in the salivary glands of *T. yao*. The mRNA transcription levels of cecropin-TY1 were dramatically up-regulated upon Gram-negative bacteria challenge at different time course (Fig. 1), suggesting that cecropin-TY1 might involve in the inflammatory response induced by the release of LPS during Gram-negative bacteria infection. In LPS-stimulated mouse peritoneal macrophages, cecropin-TY1 showed strong anti-inflammatory effects by inhibiting the transcription of iNOS and pro-inflammatory cytokines including TNF-α, IL-1β and IL-6, as well as the production of NO and these pro-inflammatory cytokines (Fig. 2 & Fig. 3). Cecropin-TY1 showed no cytotoxicity toward mouse macrophages at the concentration as high as 200 μg/mL, suggesting that its anti-inflammatory effects in LPS-stimulated macrophages were not dependent on cytotoxicity. LPS, also called endotoxin, is the characteristic components of the cell wall of Gram-negative bacteria. LPS acts as a strong stimulator to activate the innate immune system of cells, which are components of the innate immunity of diverse organisms [41, 44, 45]. Release of LPS during Gram-negative bacteria infections triggers the production of higher concentration of systemic pro-inflammatory cytokines and NO, and results in sepsis. The excessive production of systemic pro-inflammatory cytokines and NO called cytokines storm, which are responsible for the pathophysiology of septic shock and other immune diseases [46]. To address the anti-inflammatory mechanisms, the effects of cecropin-TY1 on LPS-triggered inflammatory pathways were investigated as illustrated in Fig. 4. Cecropin-TY1 significantly inhibited the activation (phosphorylation) of MAPKs and NF-κB signals induced by LPS in mouse peritoneal macrophage. These results suggested that cecropin-TY1 exerted its anti-inflammatory effects via blockade the activation (phosphorylation) of MAPKs and NF-κB signal pathways, which in turn suppressed iNOS and pro-inflammatory cytokines mRNA transcription, finally reduced NO and pro-inflammatory cytokines production.

A majority of anti-inflammatory peptides are also known to have high LPS-binding affinity and LPS-neutralizing activity such as papiliocin, cecropin A, LL-37, SMAP-29, and fowlicidin-1. These anti-inflammatory peptides can interact with LPS through binding to LPS and neutralizing LPS [29, 30, 43, 47, 48]. To clarify whether cecropin-TY1 could interact with LPS, we monitored the fluorescence change after the incubation of FITC-conjugated LPS with cecropin-TY1. As indicated in Fig. 5a, the addition of cecropin-TY1 caused a dose-dependent increase in FITC-conjugated LPS fluorescence, implying that the interaction between cecropin-TY1 and LPS resulted in the dissociation of large LPS aggregates into smaller sizes. The interaction of cecropin-TY1 with LPS was further evaluated by TAL assay, and cecropin-TY1 showed a reduced activation of LPS-induced TAL enzyme in a dose-dependent manner, suggesting that the addition of cecropin-TY1 caused the neutralization of LPS (Fig. 6b). The CD spectra indicated that cecropin-TY1 adopted a random coil structure in aqueous solution (H_2_O), but it converted to α-helical structures in the hydrophobic and/or negatively charged environments including TFE/water solution, LPS/water solution, and SDS/water solution (Fig. 6 and Table 2). The structural characterizations were further investigated by 3D structure homology modeling. It revealed that cecropin-TY1 adopted two α-helical regions in residues Leu3-Thr24 and Ile27-Leu38 linked by a hinge region (Leu25-Pro26) (Fig. 7). Like papiliocin (37 residues, helical regions in residues 3–21 and 25–36) from *P. xuthus*, cecropin A (37 residues, helical regions in residues 5–21 and 24–37) from *H. cecropia* and sarcotoxin-IA (39 residues, helical region in residues 3–23), α-helical confirmations are amphipathic structures which are responsible for the interaction of cecropins with LPS [30, 49, 50]. The same situation as cecropin-TY1, papiliocin also adopted a random coil structure in aqueous solution but converted to α-helical structure in the presence of LPS micelles, and the N-terminal amphiphilic α-helix (residues 3–18) of sarcotoxin-IA was formed upon interaction with micelles [30, 50]. In addition to amphiphilic interaction, electrostatic interaction also plays key roles in the interaction between such anti-inflammatory peptides and LPS micelles [30, 40]. There are 4 Lys residues and 2 Arg residues in the amphipathic helix in the N-terminus of cecropin-TY1, which may involve in the electrostatic interaction of cecropin-TY1 with anionic LPS (Table S2 and Fig. 7a). The electrostatic surface analyzed by 3D structure homology modeling revealed that its surface was partly positively charged (Fig. 7c). The C-terminal amidation of cecropin is important for its interaction with liposome [51]. Cecropin-TY1 is also amidated at C-terminus, which may contribute to its interaction with LPS. Besides, aromatic residues like Trp2 in N-terminus are required for the interaction of cecropin peptides with LPS [52, 53]. The N-terminus of cecropin-TY1 localized Trp2 residue, which is possibly important for its interaction with LPS (Table S2). The presence of Gly and Pro residues in the hinge region is important for the flexibility of the hinge [54]. Thereafter, the presence of Pro26 in the hinge between two helical regions may contribute to the flexibility and bending potential in the central part of cecropin-TY1 that allows the hydrophobic α-helix of the N-terminus and C-terminus to interact deeply with LPS. The mutation of these predicted structures/residues indicated that the derivatives were less active than cecropin-TY1 (Fig 3c, Fig 4g–i, Fig 6c), suggesting that these key structures/residues are crucial for the anti-inflammatory activity of cecropin-TY1.

Cecropin-TY1 was demonstrated to be a potent cecropin antimicrobial peptide in previous report [5]. In vertebrates, insects, and plants, antimicrobial peptides play pivotal roles in contribution to host defense against infections by pathogenic microorganisms. In insects, the cecropin AMPs constitute a large family of cationic α-helical peptides with antimicrobial activities against Gram-positive bacteria, Gram-negative bacteria, fungi, parasites and HIV-1 virus [20]. However, anti-inflammatory effects of cecropin family AMPs and their associated mechanisms are comparatively less investigated. As far as we know, the anti-inflammatory activities and the underlying mechanisms of insect-derived cecropin peptides have not been extensively investigated except papiliocin and cecropin A. Papiliocin and cecropin A are naturally occurred in either fat bodies or hemocytes and then release into the hemolymph [21, 30]. But no cecropins with anti-inflammatory effects were identified in salivary glands of any insects. The present work provide the first investigation of anti-inflammatory effects of cecropin antimicrobial peptide (cecropin-TY1) and its associated mechanisms of action from the salivary glands of *T. yao*.

Due to the special feeding pattern, horseflies have many chances to be infected with various microorganisms. Such defensive peptides in the salivary glands of horseflies can facilitate them to kill the microorganisms in blood meal, and protect them from pathogenic infection during feeding [5]. It has been reported that female horseflies required large amounts of blood (up to 0.5 mL) for egg production. Approximately ten feeding episodes on a host are essential for horseflies to complete a blood meal, and one landing point lasts for 3–5 min [55, 56]. The multiple landings points, continued landing time and substantial amounts of blood meal of horseflies on a host suggest that they must possess diverse potent strategies to overcome the immune response of host [55]. Not surprisingly, horseflies have evolved various countermeasures to affect the host immune response. So far, a total of 17 immunosuppressive peptides belonging to immunoregulin family have been identified and characterized from salivary glands of the horseflies *T. yao*, *Hybomitra atriperoides* and *Tabanus pleskei*, respectively [5, 18, 19]. These immunosuppressive peptides are highly conserved and composed of 30 or 35 amino acid residues. All the immuregulins exerted a decreased effect on IFN-γ and/or MCP-1 production but an increased effect on IL-10 production in LPS-stimulated mouse splenocytes. Among these immunosuppressive peptides, up to 12 members (tabimmuregulins 1–12) were identified from the salivary of *T. yao* [5]. In addition to the immunosuppressive effects of immuregulins, perhaps the anti-inflammatory effect of cecropin-TY1 is another strategy of horsefly to affect the immune response of host. As a result of co-evolution, cecropin-TY1 is possibly a potent molecule that the horsefly of *T. yao* has developed to protect their host from pathogen infection and pathogen-induced inflammatory response during blood sucking.

## Conclusion

Taken together, a potent anti-inflammatory peptide, cecropin-TY1, was identified from the horsefly salivary glands of *T. yao*. Cecropin-TY1 was demonstrated to interact with LPS and neutralize LPS, which in turn endowed cecropin-TY1 with strong anti-inflammatory effects in LPS-induced mouse macrophages without cytotoxicity. These properties make cecropin-TY1 a potential peptide candidate for the future treatment of sepsis and endotoxin shock caused by Gram-negative bacterial infections. The results also reveal a novel hint for understanding the ectoparasite-host interaction between horsefly and their host, and more work does need to be eventually done to show the true role of this peptide in the saliva in future.

## Additional file

Additional file 1: Table S1. Primers for Q-PCR. **Table S2.** Primary structural and biochemical characteristics of cecropin-TY1. (DOCX 14 kb)
