# Supplementary material for: A potent anti-inflammatory peptide from the salivary glands of horsefly
Source: Parasit Vectors. 2015 Oct 24;8:556. doi: 10.1186/s13071-015-1149-y (PMC4619319; doi:10.1186/s13071-015-1149-y)
Supplement: Additional file 1: Table S1. — Primers for Q-PCR. Table S2. Primary structural and biochemical characteristics of cecropin-TY1. (DOCX 14 kb) [file 13071_2015_1149_MOESM1_ESM.docx]

**A potent anti-inflammatory peptide from the salivary glands of horsefly.**

Lin Wei^1¶^, Chunjing Huang^1¶^, Hailong Yang^2^, Min Li^1^, Juanjuan Yang^3^, Xue Qiao^4^, Lixian Mu^2^, Fei Xiong^1^, Jing Wu^2^*, and Wei Xu^1^*

**Supplemental materials**

**Table S1. Primers for Q-PCR.**

| Primer | Forward (5’→3’) | Reverse primer (5’→3’) |
| --- | --- | --- |
| TNF-α  IL-1β  iNOS  IL-6  GAPDH  cecropin-TY1  *β*-actin | CGGTGCCTATGTCTCAGCCT  ATGGCAACTGTTCCTGAACTC  CTGCAGCACTTGGATCAGGAACCT  AGTTGCCTTCTTGGGACTGA  GTGAAGGTCGGTGTGAACGGATT  ATACGCCTTCGTTTTGGTTGT  TGTTGTCACTGTACGCCTCCG | GAGGGTCTGGGCCATAGAAC  GCCCATACTTTAGGAAGACA  GGAGTAGCCTGTGTGCACCTGGA  TCCACGATTTCCCAGAGAAC  GGAGATGATGACCCTTTTGGCTC  CACCTTGAGCAATCGGGAG  TGATGTCGCGAACGATTTCCC |

**Table S2. Primary structural and biochemical characteristics of cecropin-TY1.**

| Amino acid sequence  Number of amino acids  Net charges  Measured molecular weight  Calculated molecular weight  Theoretical pI | GWLKKIGKKIERVGQNVRNAAISTLPIAQGAAGVAGALN-NH_2_  39  +5  3970.22 Da  3970.66 Da  11.17 |
| --- | --- |
